# HIV prevention and care as part of universal health coverage

**DOI:** 10.2471/BLT.19.249854

**Published:** 2020-02-01

**Authors:** Susan P Sparkes, Joseph Kutzin

**Affiliations:** a Department of Health Systems Governance and Financing, World Health Organization, avenue Appia 20, 1211 Geneva 27, Switzerland.

The United Nations General Assembly adopted the Political Declaration of the High-Level Meeting on Universal Health Coverage (UHC) on 10 October 2019, marking the culmination of concerted efforts to bring the global health community together under a single umbrella.^1^ The UHC movement is an opportunity to consolidate disease- and intervention-specific agendas, priorities and approaches that have often led to fragmented health systems, particularly in low-income and lower-middle-income countries, where external assistance plays a key role in funding health sectors.^2^^,^^3^

The core UHC concepts of universality, non-discrimination, quality, access and protection from financial hardships are all directly relevant to the entire range of HIV services.

In this issue, Holmes et al.^4^ show the increasing convergence in the understanding of HIV services within the context of the overall UHC agenda. These efforts to show coherence are critical to developing more cohesive and patient-centred approaches to financing and service delivery for health overall and for HIV services more specifically. However, the targets of the sustainable development goals are to be achieved within ten years and therefore, it is time to move from rhetoric and concepts to action. This action should transform global commitments into country-tailored approaches, priorities and support centred on coverage and outcomes.

Concrete actions can be taken to build from the relative strengths of the UHC and HIV movements. First, the principle of universality needs to be consolidated. As countries look towards more integrated models of care, while also transitioning away from external assistance for HIV, they need to sustain and expand the coverage gains that have been made. Given that UHC is about effective coverage of services, not about a particular scheme, programme, benefit package or spending target, efforts towards financial or programmatic integration should focus on ensuring universal access.^5^ From a health ministry perspective, these efforts can include integration of HIV services into primary health care, mechanisms to contract with nongovernmental organizations to provide services to marginalized and vulnerable groups or coordination with specialized HIV delivery sites.^6^ Effective service coverage requires tailored delivery strategies for different populations and conditions, and microtargeting for HIV provides a good example.

Second, given the public-health importance of HIV and the need to ensure universal access to services, dedicated funding from government or external sources might be required. However, this requirement does not imply a need for separate, parallel subsystems, such as supply chains and information systems, nor for separate inputs such as health workers and health facilities. For countries to make the most efficient use of health-sector resources to sustain increased effective coverage of HIV services, efforts are needed to consolidate these subsystems and inputs.^7^ Countries and the partners that support them will need to engage at the level of the entire health system to leverage and build the areas of convergence. 

As Holmes et al. note, these areas include clinical platforms, health-worker performance, information systems, laboratory systems, community delivery systems and supply chain management.^3^ The focus is first on the services that need to be delivered and then on aligning inputs and financing behind those objectives, rather than letting financing sources determine service delivery organization. In the case of HIV services, the parallel functional inputs are often driven by dedicated financing coming from external assistance.^8^ Recognizing these financial incentives is one step; another is changing systems to strengthen health systems that can support HIV interventions, as well as others needed to improve the health and well-being of populations.^9^^,^^10^

Finally, just as UHC is ultimately a political issue, so too is the agenda to better align HIV investments within that context.^11^ Political issues underpin much of the sustainability agenda around HIV interventions, particularly as donor funds decrease in many contexts.^12^ Aligning programmatic approaches for HIV services with both UHC and sustainability agendas will require concerted action from global- and country-based decision-makers and organizations. Political action is needed to advocate for the importance of targeted services and interventions for HIV as part of coverage goals, in particular for key populations, as well as against continued investments in parallel inputs and systems that threaten the sustainability and effectiveness of investments in HIV services.
